# Chemical and Bioactive Properties of Red Rice with Potential Pharmaceutical Use

**DOI:** 10.3390/molecules29102265

**Published:** 2024-05-11

**Authors:** Eugénia Baptista, Ângela Liberal, Rossana V. C. Cardoso, Ângela Fernandes, Maria Inês Dias, Tânia C.S.P. Pires, Ricardo C. Calhelha, Pablo A. García, Isabel C.F.R. Ferreira, João C.M. Barreira

**Affiliations:** 1 Centro de Investigação de Montanha (CIMO), Instituto Politécnico de Bragança, Campus de Santa Apolónia, 5300-253 Bragança, Portugal; eusanba@gmail.com (E.B.); angela.liberal@ipb.pt (Â.L.); rossana@ipb.pt (R.V.C.C.); afeitor@ipb.pt (Â.F.); maria.ines@ipb.pt (M.I.D.); tania.pires@ipb.pt (T.C.S.P.P.); calhelha@ipb.pt (R.C.C.); iferreira@ipb.pt (I.C.F.R.F.); 2 Departamento de Ciencias Farmacéuticas, Facultad de Farmacia, Instituto de Investigación Biomédica de Salamanca-Centro de Investigación de Enfermedades Tropicales de la Universidad de Salamanca (CIETUS-IBSAL), University of Salamanca, 37007 Salamanca, Spain; pabloagg@usal.es; 3 Laboratório Associado para a Sustentabilidade e Tecnologia em Regiões de Montanha (SusTEC), Instituto Politécnico de Bragança, Campus de Santa Apolónia, 5300-253 Bragança, Portugal

**Keywords:** red rice, super-food, nutritional value, chemical characterization, bioactive compounds

## Abstract

Red rice has been proposed as a super-food. Accordingly, the nutritional properties (AOAC), as well as its chemical composition, including sugars (HPLC-RI), organic acids (UFLC-PDA), tocopherols (HPLD-FD), and phenolic compounds (LC-DAD-ESI/MSn), together with the main bioactive properties (antioxidant, cytotoxic, antiproliferative, and antibacterial activities), were evaluated to access its nutritional benefits and health improvement potential. The most abundant macronutrients found were carbohydrates (87.2 g/100 g dw), proceeded by proteins (9.1 g/100 g dw), fat (2.6 g/100 g dw), and ash (1.1 g/100 g dw). Sucrose and raffinose were the only detected sugars, with sucrose presenting the maximum concentration (0.74 g/100 g dw). MUFAs and PUFAs were the predominant fatty acids (40.7% and 31%, respectively). Among the two detected tocopherol isoforms, γ-tocopherol (0.67 mg/100 g dw) predominated over α-tocopherol. The phenolic compounds profile, majorly composed of flavan-3-ols, should be associated with the detected bioactivities, which may provide biological benefits to human health beyond the primary nutritional effect. Overall, the bioactive potential of red rice was comprehensively accessed.

## 1. Introduction

Established as a primary food of high nutritional value, rice (*Oryza sativa* L.) is a whole grain widely consumed around the world by more than half the population, especially in Asia, the continent where approximately 95% of rice is produced. In several parts of the globe, this cereal is one of the primary elements of a regular diet, both through human consumption and ultimately as animal feed, making it one of the highly valuable cereals around the world [1]. The nutritional quality of rice comes from its richness in carbohydrates and its moderate amounts of crude protein and fat, being also a basis of vitamin B complexes, such as niacin, riboflavin, and thiamine, and minerals such as calcium (Ca), magnesium (Mg), and phosphorus (P), along with minor amounts of copper (Cu), iron (Fe), manganese (Mg), and zinc (Zn) [1,2]. The fact that some cereal brans, such as rice, are rich in bioactive compounds, dietary fibers, essential fatty acids, phytosterols, antioxidants, among others already mentioned, make these cereals widely important food products since such attributes have positive effects on human health [3]. In recent years, pigmented rice varieties have been attracting great attention from consumers, given the global demand for healthy foods (such as high-quality rice), their composition in bioactive phenolic compounds, tocols, and sterol derivatives, and their potential health benefits (e.g., antioxidant and anti-inflammatory properties) [4]. In traditional Asian cultivation areas, different varieties of rice are known and cultivated, including red, purple, black, brown, yellow, and green rice; these designations refer to the color of the kernel, which include different compounds such as phenolic acids, anthocyanins, and proanthocyanidins in different layers of the pericarp (bran layer, integument, and aleurone) [5]. Red rice owes its color to the existence of proanthocyanidins [6] located in the bran coating, which are responsible for its higher antioxidant capacity compared to other rice varieties. It is also a fact that red-grained rice holds an exclusive taste and a superior nutritional quality and health benefits, the reason why its consumption has been preferred over the years [7].

Several studies have focused on the health benefits arising from red rice consumption, highlighting its antioxidant, antidiabetic, anti-inflammatory, and anti-neurodegenerative potential, among others, as well as its noteworthy contribution in obesity and obesity-related diseases prevention [8]. In addition, due to its gluten-free nature, this cereal has been widely used by intolerant people, including babies and children [9], serving as a basis for the preparation of infant formula and baby food [9,10]. Red rice has also been applied in the manufacture of bread and colored pasta and in the cosmetic industry, among others [11,12].

Despite its valuable characteristics, which include not only a rich nutritional value but also a wide range of natural bioactive compounds with positive impacts on human health, red rice is generally a poorly consumed and undervalued variety. Thus, this work aims to evaluate the nutritional and chemical profiles of red rice samples grown in Cambodia, as well as its phenolic composition and bioactive properties, to better characterize this variety of rice and boost its introduction into a balanced and healthy diet. The red rice crop is photosensitive, and in Cambodia, it is planted in June and harvested in December with only 1 harvest per year, while round and long rice from the same country is harvested 2–3 times a year. This type of rice requires fewer fertilizers and chemicals as it is a more resistant species than conventional rice, being planted in lowlands.

## 2. Results and Discussion

The described tests were performed in triplicate and the results expressed as mean values ± SD.

### 2.1. Nutritional and Chemical Composition of Red Rice

Information regarding the nutritional value of the analyzed red rice sample is displayed in Table 1. Carbohydrates were the main macronutrient in red rice, with a mean concentration of 87.2 g/100 g dw. Proteins, in turn, stood out as the second major nutrient (9.1 g/100 g dw), which agrees with a study performed earlier by Van Hung et al. [13]. Likewise, another study [14] also shows similar amounts of carbohydrates (80.20 g/100 g dw) and proteins (8.47 g/100 g dw). Lastly, red rice presented low fat amounts (2.6 g/100 g dw) and mineral compounds, given the small ash content, which is slightly above 1 g/100 g dw. Sompong et al. [15] described an even smaller amount of fat and ash between different varieties of red rice, with mean concentrations of 1.17 and 0.98 g/100 g dw, respectively, for the Sri Lanka variety. However, Vargas et al. [14] and Somaratne et al. [16] identified similar amounts of fat (2.67 and 2.4 g/100 g dw) and ash (1.82 and 1.44 g/100 g dw). These differences and similarities may be related to edaphoclimatic factors, affecting their nutritional and chemical composition. In general, lipids are highly associated with the preservation and quality of stored cereals and flours, being considered as an important nutritional and physiological parameter [17].

Sucrose and raffinose were the only identified free sugars (Table 1), both in low concentrations, but with a greater prevalence of sucrose (0.740 g/100 g dw) over raffinose (0.38 g/100 g dw).

In what concerns the organic acid profile (Table 1), oxalic, citric, and fumaric acids could be detected, although in trace amounts. To the best of our expertise, this is the first description of soluble sugar and organic acid identification in red rice samples.

Fatty acid and tocopherol profiles are also stated in Table 1. Thirteen fatty acids were identified, with a clear dominance of oleic (C18:1n9c, 39.9%) and linoleic acids (C18:2n6c, 29.4%). Among the saturated fatty acids, palmitic acid (C16:0, 22%) showed the highest concentration, followed by stearic acid (C18:0, 3.0%). Since essential fatty acids (omega-6 and omega-3 families) are mainly found in the composition of plant cells such as rice, and cannot be produced by the human organism, the inclusion of food products with substantial concentrations of these compounds in the daily diet must be promoted. In general, the analyzed red rice sample showed higher amounts of monounsaturated fatty acids (MUFAs, 40.7%), followed by polyunsaturated fatty acids (PUFAs, 31%) and saturated fatty acids (SFAs, 28.4%). PUFAs hold critical functions in human growth and in the delay and treatment of illnesses such as hypertension, heart disease, diabetes, arthritis, cancer, and other inflammatory and autoimmune diseases [18]. Even though the amount of MUFA and PUFA present in rice is considerably small when compared to other foods, such as nuts, its inclusion in a daily diet may contribute to the overall supply of these type of fatty acids.

Lastly, α-tocopherol (0.23 mg/100 g dw) and γ-tocopherol (0.67 mg/100 g dw) were the only detected isoforms. However, Yu et al. [19] and Shen et al. [20] described smaller quantities (despite being as well the main isoform) of γ-tocopherol (0.317–0.413 mg/100 g dw) than those quantified in our study. The same authors, in a related study, reported significantly higher values of the γ-tocopherol isoform (1.026 mg/100 g dw) [19], which indicates the possible influence of other factors (mainly edaphoclimatic) besides genetic ones.

### 2.2. Phenolic Profile

The chromatographic and spectral characteristics (retention time (Rt), maximum absorption wavelengths in the visible region (λ_max_), and mass spectral data), tentative identification, and quantification data (mg/g of extract) of the phenolic compounds present in the hydroethanolic extracts of the red rice sample are presented in Table 2. Nine phenolic compounds were tentatively identified: three phenolic acids (caffeic, ferulic, and sinapic acids) derivatives and six flavan-3-ols (catechin derivatives).

In what concerns phenolic acids, peak 1, tentatively identified as caffeic acid hexoside, presents a pseudomolecular ion [M-H]^−^ at *m*/*z* 341 and MS^2^ fragments at *m*/*z* 179, 161, and 135, which represented the breaking of the unit of caffeic acid and the loss of a hexosyl portion (341 − 179 = 162 u) [21]. Peak 4, which showed a pseudomolecular ion [M-H]^−^ at *m*/*z* 517 and λ_max_ 322 (characteristic of ferulic acid derivatives), was tentatively identified as ferulic acid dihexoside, as previously described by Zaupa et al. [22] in pigmented rice varieties. Finally, peak 9 was tentatively identified as sinapoyl feruloyl dihexoside ([M-H]^−^ at *m*/*z* 723 and λ_max_ 322), comparing its chromatographic characteristics with the previously described ones by Pacifico et al. [23] in wild rue hydroalcoholic extracts. An in-depth study of the occurrence of phenolic acids in red rice was reported by Li et al. [24], which not only identified ferulic and caffeic acids, as in our study, in their samples but also gallic, protocatechuic, vanillic, and syringic acids, among others, which may be related not only to the variety but also to the different origins of the samples and,= consequently to the different edaphoclimatic conditions to which they were exposed.

Flavan-3-ols embodied the bulk of the detected phenolic compounds in the analyzed red rice sample. Peaks 2, 3, 5, 6, 7, and 8 correspond to proanthocyanidins based on the presented pseudomolecular ion and fragmentation pattern in MS^2^. The information obtained by mass spectrometry allowed the identification of the elementary units constituting the molecules as also their relative position but did not allow the identification of the position between the (epi)catechin units and the differentiation between the isomers of catechin. As such, the peaks have been tentatively identified as a type B catechin dimer (peak 2), trimer (5, 6, and 8), and tetramer (peaks 3 and 7) based on the previously described ones by Gu et al. [25] and Zaupa et al. [22] in different rice varieties.

Data regarding the phenolic compounds’ quantification show that, relative to phenolic acids, ferulic acid dihexoside was the main compound (0.093 mg/g of extract) found in red rice. In turn, Lang et al. [26] reported ferulic acid in higher quantities (0.3159 mg/g of extract), being similarly the main compound observed in their study. However, Ziegler et al. [27] reported lower amounts of ferulic acid (3.8 ± 0.3 µg/g of extract) in a Brazilian variety of red rice than we found in our sample, showing the impact of different soil types, climates, and other factors on the chemical composition of cereals such as red rice.

As previously mentioned, the flavan-3-ol group represented most of the compounds found in the red rice sample, more specifically, ≈98% (5.1 mg/g of extract of the total phenolic compounds). Among these, the main compounds found were a type B (epi)-catechin dimer (peak 2: 2.22 mg/g of extract), followed by a type B (epi)-catechin trimer (peak 6: 1.03 mg/g extract) and a type B (epi)-catechin tetramer (peak 3: 0.83 mg/g extract). Other authors have also identified catechin and its derivatives in red rice samples [26,27,28]. The phenolic compound profile of red rice was previously considered by several authors, with distinct varieties being analyzed and different phenolic compounds and respective mean concentrations reported [16,19,26].

### 2.3. Bioactive Properties

Phytochemicals in whole grain rice are mainly found in the bran layer, composed of lipophilic (tocopherols and tocotrienols) and insoluble compounds [24]. Red rice covers a diversity of bioactive compounds with beneficial health effects, with studies showing that pigmented rice tocopherols may hold some anticancer properties and may hinder the development of cancer cells [29,30]. Phenolic compounds, for instance, have been broadly investigated, especially for their biological properties as natural antioxidants, contributing to a reduced risk of developing chronic diseases related to oxidative stress, cardiovascular diseases, and neural diseases [8]. The antioxidant activity of the hydroethanolic extract of red rice was evaluated through the determination of their aptness to suppress lipid peroxidation, and the results are described in Table 3. The red rice sample revealed an EC_50_ value of 0.51 mg/mL, which was not comparable to those of other matrices with higher antioxidant activity. Several studies have reported the antioxidant activity in different red rice varieties, using different in vitro chemical methods [31,32], reporting uneven results that might be due to the low reproducibility and different experimental parameters that are typically associated with these methods. It has already been established that rice bran contains higher phenolic content than wheat bran [33], and these secondary metabolites are mostly identified in pigmented rice. These compounds are known to have an antioxidant capacity and act as metal ion chelators, free radical scavengers, and reducing agents [31]. Thus, the scavenging of free radicals by the bioactive compounds present in whole rice grains maintains their integrity, protecting them from adverse effects caused by reactive oxygen species (ROS). Among these bioactive compounds, flavan-3-ols stand out as the group of phenolic compounds with the greatest antioxidant power, providing several benefits to human health. Accordingly, the literature has revealed that bran from red and black rice cultivars has higher phenolic content and antioxidant activity than bran from non-pigmented rice varieties [34].

In terms of antiproliferative properties, the most sensitive cell lines to red rice hydroethanolic extracts were lung carcinoma (NCI-H460: 205 mg/mL) and liver carcinoma (HepG2: 291 mg/mL); MCF-7 and HeLa presented higher GI_50_ values which translated to the reduced efficacy of the extracts against these tumor lines. The phenolic compounds found in red rice may have been partially responsible for the results obtained in NCI-H460 and HepG2. Similarly, a local pigmented rice cultivar from Thailand called KumPhayao was noticed extremely cytotoxic to human hepatocellular carcinoma HepG2 cells when compared with other Northern Thai purple rice varieties, which seems to be related to the high anthocyanin contents found in the analyzed variety (cyanin 3-glucosides and peonidin-3-glucosides). This study shows that a methanolic extract of the KumPhayao pigmented rice variety induced human HepG2 cell apoptosis via the mitochondrial pathway [35].

Some of the compounds present in pigmented rice may contain chemopreventive agents, a fact proven by Guo et al. [36], who detected the anti-mutagenic effects of cooked glutinous purple rice extract (*O. sativa* L. var. indica) on diethylnitrosamine (DEN)-induced hepatic preneoplastic lesions in rats. Also, another study revealed that a methanol extract of purple rice (*O. sativa* L.). cv. KumPhayao bran collected from Thailand contained high amounts of phenolic compounds and exhibited cancer chemopreventive effects on the AFB1-induced initiation stage of hepatocarcinogenesis in rats [37], supporting the idea that pigmented rice varieties can strongly inhibit tumor promoters compared to non-pigmented varieties.

Data regarding hepatotoxicity show that the analyzed sample did not show cytotoxicity against pig liver cell lines (PLP2).

Lastly, the results attained for the antibacterial activity of the red rice sample are shown in Table 4. The sample was analyzed in opposition to Gram-positive (*Enterococcus faecalis*, *Listeria monocytogenes*, and MRSA) and Gram-negative bacteria (*Escherichia coli*, *Klebsiella pneumoniae*, *Morganella morganii*, *Proteus mirabilis*, and *Pseudomonas aeruginosa*) and compared with positive controls (ampicillin, imipenem, and vancomycin). However, red rice extracts showed weak antibacterial activity, presenting minimum inhibition concentration (MIC) values of 10 mg/mL for Gram-positive bacteria and 20 mg/mL (or higher) for Gram-negative bacteria; in all cases, the minimum bactericidal concentration (MBC) values were higher than 20 mg/mL, which should be considered as a desired feature when working with a food product. The antibacterial activity of rice was previously tested in its by-products; yet, the bacteria strains used also showed little or practically no sensitivity to the tested extracts [38]. To the best of our understanding, this is the first assessment of the antibacterial activity of red rice. However, Călinoiu and Vodnar [39] previously reported moderate antibacterial activity in a thermally processed wheat bran sample (e.g., an MIC of 1.875 mg/mL against *E. faecalis* and an MIC of 3.75 mg/mL against *E. coli*), which, as it is also a cereal, can be somehow compared to rice.

Although there are already some studies on the nutritional and chemical profiles and bioactive capacity of red rice, these are not significant in the sense that the vast majority do not exhaustively describe and/or group the different parameters analyzed in this study. Additionally, this research is relevant as it analyzes a variety of rice from Cambodia, which is therefore subject to distinctive edaphoclimatic conditions that influence the physicochemical and bioactive profile of agricultural products and may constitute a starting point for the study of these variabilities in this and other types of products.

## 3. Materials and Methods

### 3.1. Samples

The rice sample belongs to the indica variety, and it was produced in Cambodia. The pericarp was preserved since this was the structure in which the pigments that give it the characteristic red color of this type of rice are found. Their analysis was preceded by grinding the rice into a fine powder (100 mesh), being subsequently stored at room temperature in food-grade plastic bags protected from light and humidity. Rice was processed with low kett (whole grain) to preserve the anthocyanins found in the bran and the rest of the nutrients that are lost when polishing the rice.

### 3.2. Extract Formulation

Hydroalcoholic extraction (ethanol/water, 80:20 *v*/*v*) was made by stirring the dry samples for the investigation of the phenolic profile and other bioactive compounds; concisely, 1 g of the sample was extracted twice (1 h, 25 °C, 150 rpm in each step) through a 1:30 solid-to-liquid ratio and then filtered (Whatman No. 4 paper). Subsequently, the ethanolic fraction was evaporated employing a rotary evaporator (Büchi R-210, Flawil, Switzerland), and samples were ice-covered and freeze-dried (FreeZone 4.5 model 7750031, Labconco, Kansas City, MO, USA) for the subsequent evaluation [40].

### 3.3. Basic Nutritional Value

The red rice was tested for its content in fat, proteins, and ash by applying the official methods of analysis (AOAC) [41]. The macro-Kjeldahl procedure was used in the determination of crude protein content (*N* × 5.95) using an automated distillation and titration unit (model Pro-Nitro-A, JP Selecta, Barcelona, Spain). The ash content was accessed via the incineration of the dried samples at 550 ± 15 °C. Crude fat was established through a Soxhlet system through the extraction of a known mass of the red rice sample with petroleum ether, and the results were expressed in g/100 g of dry weight (dw). Total carbohydrates were estimated by difference: total carbohydrates (g/100 g dw) = 100 − (g protein + g fat + g ash). The energetic value was determined from the Atwater equation: energy (kcal/100 g dw) = 4 × (g protein + g carbohydrates) + 9 × (g fat). All tryouts were conducted in triplicate, and the outcomes were stated as mean values ± standard deviation (SD). Moisture in each sample (2 g) was quantified using a moisture analyzer (Adam Equipment, PMB 163, Kingston, UK).

### 3.4. Chemical Characterization

#### 3.4.1. Free Sugars

Free sugars extraction was performed according to a procedure previously described by Barros et al. [40], and compounds were set by high-performance liquid chromatography linked to a refraction index detector (HPLC-RI) (HPLC-RI; Knauer, Smartline 1000 and RI—Knauer, Berlin, Germany, respectively) [42]. Briefly, the chromatographic separation was achieved with a Eurospher 100-5 NH2 column (4.6 × 250 mm, 5 mm, Knauer) operating at 30 °C (7971 R Grace oven). The mobile phase was acetonitrile/deionized water, 70:30 (*v*/*v*), with a flow rate of 1 mL/min. The molecular identification was made through a comparison of their retention times (Rt) along with the original standards and quantification applying melezitose as the internal standard (IS). The findings were stated and acknowledged using the Clarity 2.4 software (DataApex, Podohradská, Czech Republic) and expressed in g/100 g of dw.

#### 3.4.2. Organic Acids

The assessment of organic acids, as earlier defined by the authors [42], was made via ultra-fast liquid chromatography combined with a photodiode array detector (UFLC-PDA; Shimadzu Corporation, Kyoto, Japan), and the chromatographic separation occurred in a C18 SphereClone (Phenomenex, Torrance, CA, USA) reverse-phase column (5 µm, 250 × 4.6 mm i.d.) thermostated at 35 °C, utilizing 3.6 mM sulfuric acid solution as an eluent at a flow rate of 0.8 mL/min. The classification was performed by making a comparison of the chromatograms obtained for the analyzed samples with commercial standards. The quantification of organic acids was carried out by relating the peak areas recorded at 215 nm, with the calibration curves obtained with authentic commercial standards for each compound. The outcomes were stated in g/100 g dw.

#### 3.4.3. Fatty Acids

The lipid fraction obtained through Soxhlet extraction was subjected to a trans-esterification process to obtain the fatty acid methyl esters (FAME), as formerly explained by Barros et al. [40], and defined by gas–liquid chromatography with flame ionization detection, employing a YOUNG IN Crhomass 6500 GC System instrument supplied with a split injection 1/80, a flame ionization detector (FID), and a Zebron-Fame column. Fatty acid identifications and quantifications were performed by comparing the relative retention times of FAME peaks from samples with standards, and the results were logged and treated using the Clarity DataApex 4.0 software (Prague, Czech Republic) and articulated in comparative percentages of individual fatty acids.

#### 3.4.4. Tocopherols

Tocopherols were evaluated through a process earlier explained by the authors [43]. The formerly termed HPLC system, linked to a fluorescence detector (FP-2020; Jasco, Tokyo, Japan) designed for excitation at 290 nm and emission at 330 nm, was used. The separation of the tocopherol isoforms was conducted applying a regular phase column of polyamide II (250 mm × 4.6 mm i.d.) from YMC Waters (Kyoto, Japan), running at 30 °C. The mobile phase employed was a combination of hexane and ethyl acetate (70:30, *v*/*v*), with a flow rate of 1 mL/min and an injection volume of 20 µL. The isoform identification was completed by making chromatographic comparisons with the original commercial standards, and the quantification was centered on the response of the fluorescence indicator via the internal standard procedure and by chromatographic similarity with the standards. Tocol was utilized as an internal standard, and the results were stated in mg/100 g dw.

#### 3.4.5. Phenolic Compounds

The phenolic compounds were assessed in the lyophilized hydroethanolic extract (described in Section 3.2) of red rice and re-dissolved in ethanol/water (80:20, *v*/*v*) to a last concentration of 10 mg/mL. The analysis was achieved employing a Dionex Ultimate 3000 UPLC (Thermo Scientific, San Jose, CA, USA) system supplied with a diode array sensor (DAD) (280 and 370 nm as the selected wavelengths), combined with an electrospray ionization mass detector (LC-DAD-ESI/MS^n^). The chromatographic separation of the compounds was executed with a Waters Spherisorb S3 ODS-2 C18 column (3 µm, 4.6 mm × 150 mm, Waters, Milford, MA, USA) at 35 °C, as defined by Bessada et al. [44]. The elution solvents running in the gradient were 0.1% formic acid in water and acetonitrile. For detection MS in the negative mode, a Linear Ion Trap LTQ XL mass spectrometer (ThermoFinnigan, San Jose, CA, USA) provided with an electrospray ionization source (ESI) was utilized. The compounds were identified based on their chromatographic performance, UV-vis spectra, and masses via comparisons with standard compounds, when accessible, or data that were established before, through the Xcalibur^®^ software 4.0 (ThermoFinnigan, San Jose, CA, USA). The quantification of the detected compounds was completed through 7-level calibration curves built based on the UV signal of the standard compounds (caffeic acid (*y* = 388,345*x* + 406,369. *R*^2^ = 0.9939); catechin (*y* = 84,950*x* − 23,200*. R*^2^ = 1); ferulic acid (*y* = 633,126*x* − 185,462*. R*^2^ = 0.999); and sinapic acid (*y* = 197,337*x* + 30,036. *R*^2^ = 0.9997)). When no commercial standard was available, the quantification was built on calibrations produced beside the greatest related standard. The results were given in mg/g of extract.

### 3.5. Bioactive Properties

#### 3.5.1. Antioxidant Activity

The antioxidant potential of the red rice hydroethanolic extract was measured through lipid peroxidation inhibition by a thiobarbituric acid reactive substance assay (TBARS), performed using porcine brain cells (*Sus domesticus*), via subtractions, according to previously described procedures [45]. The outcomes were expressed in EC_50_ values, which represented the extract concentration (mg/mL) necessary to prevent 50% of lipid peroxidation in porcine brain cells, and trolox was used as the standard.

#### 3.5.2. Antiproliferative and Cytotoxic Activity

The antiproliferative capacity of the extracts was evaluated by the sulforhodamine B assay against four human tumor cell lines: MCF-7 (breast adenocarcinoma), NCI-H460 (non-small cell lung cancer), HeLa (cervical carcinoma), and HepG2 (hepatocellular carcinoma). The density of the cell solution used in each assay is prepared with the corresponding/indicated culture medium for each strain. Cell line cultures were prepared with RPMI medium and seeded in 96-well plates in concentrations ranging from 6.25 to 400 µg/mL, with a final density of 1.0 × 10^4^ cells/well, and fixation was permitted for 24 h. Subsequently, different concentrations of the extract were put into the cells and incubated for 48 h, as earlier described [43]. Ellipticin was utilized as a positive regulator. The hepatotoxicity of the extracts against a non-tumor cell line (PLP2, porcine liver primary cells) obtained as previously described [17] was performed using the same procedure. Outcomes were articulated as GI_50_ values, relative to the extract concentration (μg/mL) that caused 50% of cell growth inhibition. All cell lines used in this project are commercially available and were purchased from different authorized cell line resources including the German Collection of Microorganisms and Cell Cultures (DSMZ, Braunschweig, German) and the European Collection of Authenticated Cell Cultures (ECCAC, Salisbury, UK). A primary cell line obtained from porcine liver tissue (PLP2) was also prepared to study the cytotoxicity effect in non-tumoral cells. To maintain high scientific standards, all procedures were performed according to the best practices observed in the Guidance on Good Cell Culture Practice (GCCP) and authorized by the ethics commission (number 336101) of the Instituto Politécnico de Bragança (IPB).

#### 3.5.3. Antibacterial Activity

The antibacterial potential of the red rice hydroethanolic extract was evaluated for clinical isolates from different specialties of Centro Hospitalar de Trás-os-Montes e Alto Douro and Unidade Local de Bragança, Northeast Portugal, following techniques formerly explained by Pires et al. [46]. Five Gram-negative bacteria (*Escherichia coli*, *Proteus mirabilis*, *Klebsiella pneumoniae*, *Pseudomonas aeruginosa,* and *Morganella morganii*) and four Gram-positive bacteria (*Enterococcus faecalis*, *Listeria monocytogenes*, *Staphylococcus aureus,* and methicillin-resistant *Staphylococcus aureus*—MRSA) were used. The results were exhibited as the minimal inhibition concentration (MIC), stated as the lower extract concentration that displayed the whole inhibition of bacterial development, and the minimal bactericidal concentration (MBC), specified as the lowest concentration of an antibacterial mediator essential to inhibit the growth of a particular bacterium. Ampicillin was employed as a positive control for all bacterial strains, imipenem for all Gram-negative bacteria tested, and *L. monocytogenes* and vancomycin for *E. faecalis* and MRSA.

## 4. Conclusions

Through the years, pigmented rice has been investigated due to its rich composition of macronutrients and beneficial bioactive compounds such as sterols, vitamins, phenolic compounds, and others, being highlighted for having important nutritional and medicinal properties. Secondary metabolites are mostly identified in pigmented rice, which includes red rice varieties. These secondary metabolites, mainly composed by different phenolic compounds, including pigments, are predominantly present in rice bran, which make pigmented rice a more valuable source of bioactive compounds when compared with non-pigmented varieties. Our investigation into red rice, in which some results have been reported for the first time, namely soluble sugar and organic acid occurrences, as well as antibacterial activity (despite the limitation that samples of non-pigmented rice were not analyzed at the same time), allowed as well its nutritional and chemical characterization besides identifying several individual compounds, such as tocopherols, caffeic, ferulic and sinapic acids, and catechin derivatives, whose occurrence can be associated with its antioxidant and antiproliferative activities, namely against lung (NCI-H460: 205 mg/mL) and liver (HepG2: 291 mg/mL) carcinomas. Also, a slight antibacterial activity against Gram-positive species were detected for the first time. The validation of red rice as a super-food was also proven in the present study since we were able to identify a rich profile of bioactive compounds, essential fatty acids, antioxidants, among others already mentioned, attributes that allow beneficial influences on human health.

Besides their scientific contribution, the obtained results might render some benefits to the cereal processing industry as they contribute to characterizing a staple food. In fact, our results emphasize that red rice varieties should be valued, improved, and further explored as an alternative food product, contributing to the necessary and continuous effort in adding value to typically underexplored agricultural crops. Thus, further investigations in pigmented rice and its constituents, namely its content in dietary fiber and a precise and accurate analysis of its pigments, will further contribute to clarifying its nutritional value and the mechanisms underlying the usefulness of its bioactive compounds, thus improving the understanding of its various roles in human health and the food industry.

## Figures and Tables

**Table 1:** Nutritional value and hydrophilic and lipophilic compounds of the studied red rice (mean ± SD).

Nutritional Value (g/100 g dw)	Red Rice
Fat	2.6 ± 0.1
Proteins	9.1 ± 0.1
Ash	1.1 ± 0.1
Carbohydrates	87.2 ± 0.2
Energy value (kcal/100 g dw)	408.6 ± 0.1
Hydrophilic compounds	
Free sugars (g/100 g dw)	
Sucrose	0.740 ± 0.002
Raffinose	0.38 ± 0.02
Total sugars	1.12 ± 0.03
Organic acids (g/100 g dw)	
Oxalic acid	tr
Citric acid	tr
Fumaric acid	tr
Lipophilic compounds	
Fatty acids (%)	
C14:0	0.34 ± 0.01
C15:0	0.122 ± 0.004
C16:0	22 ± 1
C16:1	0.142 ± 0.004
C18:0	3.0 ± 0.1
C18:1n9c	39.9 ± 0.2
C18:2n6c	29.4 ± 0.5
C18:3n3	1.2 ± 0.1
C20:0	1.1 ± 0.1
C20:1	0.58 ± 0.03
C22:0	0.911 ± 0.001
C20:5n3	0.25 ± 0.01
C24:0	1.03 ± 0.02
SFA	28.4 ± 0.4
MUFA	40.7 ± 0.1
PUFA	31 ± 1
Tocopherols (mg/100 g dw)	
α-Tocopherol	0.23 ± 0.01
γ-Tocopherol	0.67 ± 0.03
Total tocopherols	0.89 ± 0.04

dw—dry weight; tr—traces; C14:0—myristic acid; C15:0—pentadecanoic acid; C16:0—palmitic acid; C16:1—palmitoleic acid; C18:0—stearic acid; C18:1n9c—oleic acid; C18:2n6c—linoleic acid; C18:3n3—linolenic acid; C20:0—arachidic acid; C20:1—cis-11-eicosenoic acid; C22:0—behenic acid; C20:5n3—eicosatetraenoic acid; C24:0—lignoceric acid; SFA—saturated fatty acid; MUFA—mono-unsaturated fatty acid; PUFA—polyunsaturated fatty acid.

**Table 2:** Retention time (Rt), wavelengths of maximum absorption in the visible region (λ_max_), mass spectral data, tentative identification, and quantification of the phenolic compounds in red rice hydroethanolic extracts.

Peak	Rt (min)	λ_max_ (nm)	[M-H]^−^ (*m*/*z*)	MS^2^ (*m*/*z*)	Tentative Identification	Quantification (mg/g Extract)
1	4.91	311	341	179(100), 161(15), 135(5)	Caffeic acid hexoside	0.01562 ± 0.00002
2	6.24	280	577	451(29), 425(100), 407(30), 289(15)	Type B (epi)catechin dimer	2.22 ± 0.04
3	7.48	280	1153	865(15), 577(20), 289(100)	Type B (epi)catechin tetramer	0.83 ± 0.02
4	9.88	322	517	355(55), 193(100)	Ferulic acid dihexoside	0.093 ± 0.001
5	10.89	280	865	577(50), 451(30), 425(100), 407(38), 289(20)	Type B (epi) catechin trimer	0.40 ± 0.01
6	12.05	280	865	577(48), 451(28), 425(100), 407(28), 289(15)	Type B (epi) catechin trimer	1.03 ± 0.03
7	13.93	280	1153	865(15), 577(20), 289(100)	Type B (epi) catechin tetramer	0.188 ± 0.001
8	15.23	280	865	577(52), 451(29), 425(100), 407(37), 289(15)	Type B (epi) catechin trimer	0.42 ± 0.01
9	23.42	322	723	517(34), 355(39), 337(81), 295(34), 193(100), 175(52), 161(5)	Sinapoyl feruloyl dihexoside	0.061 ± 0.001
					Total phenolic acids	0.1691 ± 0.0004
					Total flavan-3-ols	5.1 ± 0.1
					Total phenolic compounds	5.2 ± 0.1

Calibration curves used: caffeic acid (*y* = 388,345*x* + 406,369. *R*^2^ = 0.9939, peak 1); catechin (*y* = 84,950*x* − 23,200*. R*^2^ = 1, peaks 2, 3, 5, 6, 7, and 8); ferulic acid (*y* = 633,126*x* − 185,462*. R*^2^ = 0.999, peak 4); sinapic acid (*y* = 197,337*x* + 30,036. *R*^2^ = 0.9997, peak 9).

**Table 3:** Antioxidant, antitumor, and hepatotoxic activities of red rice hydroethanolic extracts (mean ± SD).

Antioxidant activity	(EC_50_ mg/mL)	Trolox (μg/mL)
TBARS	0.51 ± 0.01	19.6 ± 0.1
Antitumor activity	(GI_50_ μg/mL)	Ellipticine (μg/mL)
HeLa (cervical carcinoma)	343 ± 7	0.9 ± 0.1
NCI H460 (lung carcinoma)	205 ± 11	1.03 ± 0.09
MCF7 (breast carcinoma)	322 ± 11	1.21 ± 0.02
HepG2 (liver carcinoma)	291 ± 10	1.10 ± 0.09
Hepatotoxicity	(GI_50_ µg/mL)	Ellipticine (μg/mL)
PLP2	>400	2.3 ± 0.2

The antioxidant activity was expressed in EC_50_ values, which means that high values correspond to low antioxidant potential. EC_50_ corresponds to the concentration of extract necessary to obtain 50% of antioxidant activity. Cytotoxicity results were expressed as GI_50_ values, corresponding to the sample concentration required to perform 50% growth inhibition under human tumor cells.

**Table 4:** Antibacterial activities (mg/mL) of red rice hydroethanolic extracts (mean ± SD).

	Red Rice		Ampicillin		Imipenem		Vancomycin	
	MIC	MBC	MIC	MBC	MIC	MBC	MIC	MBC
Gram-negative bacteria								
*Escherichia coli*	20	>20	<0.15	<0.15	<0.0078	<0.0078	n.t.	n.t.
*Klebsiella pneumoniae*	20	>20	10	20	<0.0078	<0.0078	n.t.	n.t.
*Morganella morganii*	20	>20	20	>20	<0.0078	<0.0078	n.t.	n.t.
*Proteus mirabilis*	>20	>20	<0.15	<0.15	<0.0078	<0.0078	n.t.	n.t.
*Pseudomonas aeruginosa*	>20	>20	>20	>20	0.5	1	n.t.	n.t.
Gram-positive bacteria								
*Enterococcus faecalis*	10	>20	<0.15	<0.15	n.t.	n.t.	<0.0078	<0.0078
*Listeria monocytogenes*	10	>20	<0.15	<0.15	<0.0078	<0.0078	n.t.	n.t.
MRSA	10	>20	<0.15	<0.15	n.t.	n.t.	0.25	0.5

n.t.—not tested; MRSA—methicillin-resistant *Staphylococcus aureus*; MIC—minimum inhibitory concentration; MBC—minimal bactericidal concentration.

## Data Availability

Data are contained within the article.
